# Dancing of electromagnetic spectra driven by metasurfaces

**DOI:** 10.1093/nsr/nwz042

**Published:** 2019-03-27

**Authors:** Min Gu

**Affiliations:** Laboratory of Artificial-Intelligence Nanophotonics, School of Science, RMIT University, Australia

In 2011, Capasso proposed the concept of manipulating electromagnetic (EM) waves with phase modulation by an ultrathin flat metasurface composed of nanoantenna arrays [1]. Later, researchers developed different kinds of new metasurfaces, such as hyperbolic metasurfaces, tunable metasurfaces and non-linear metasurfaces [2]. The development of metasurfaces has greatly enhanced the capability to manipulate EM waves with multiple functionalities on a flat surface. Non-linear metasurfaces combine the advantages of metasurface and non-linear EM phenomena, offering new degrees of freedom for the improvement of the performance of metasurfaces. The non-linear response of metasurfaces can be used to realize functions such as frequency mixing, harmony generation and ultrafast switching [3], which are essential for classical and quantum communication. However, the strength of the non-linear EM phenomena of the metasurfaces is a naturally given constant that cannot be changed after the metasurfaces have been fabricated. Further, the efficiency of non-linear EM phenomena in metasurfaces is usually very weak due to the limitation of the non-linear materials of the metasur-faces.

The invention of digital coding metasurfaces (DCM) offers a programable way to control the EM waves with one physical configuration [4,5]. The unit cells of the DCM have a digital response (‘0’ or ‘1’) controlled by a biased diode that can be switched in the time domain. Such metasurfaces offer a new dimension to control the non-linear response with an external voltage bias. Recently, using a DCM with varying reflectivity controlled by the biased diode, scientists in China and the USA have demonstrated harmony generation with high conversion efficiency, which is not limited by the optical properties of materials [6].

**Figure 1.**
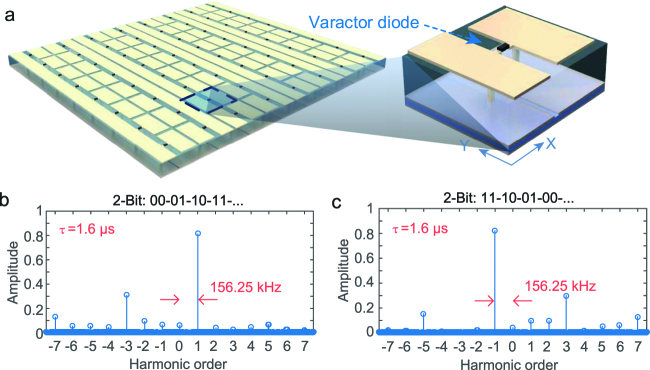
(a) Side view of the time-domain DCM with a zoomed view of the meta-atom on the right. (b, c) The measured harmonic distributions from -7th to 7th orders under two-bit coding sequences 00-01-10-11-… and 11-10-01-00-… at 3.6 GHz, respectively. (Reprint from Fig. 3 of [6].)

The team of Tie Jun Cui, Qiang Cheng and Shi Jin (Jie Zhao, Xi Yang, Jun Yan Dai, Xiang Li, Ning Hua Qi, Jun Chen Ke, Guo Dong Bai, and Shuo Liu), at Southeast University in China, and Andrea Alù, at the City College of New York, designed a reflective time-domain digital coding metasurface to control the spectra of the illuminated signal (Fig. 1). The amplitudes of the generated harmonics were closely related to the shape of the periodic reflection function. Different strategies could be employed to alter the spectral responses of the metasurfaces, including amplitude and phase modulations of the surface reflectivity. In their tests, the authors used stepped reflection phases that were encoded in each period, and the results demonstrated a significant frequency shifting, with 98.45% of the carrier energy converted to high-order harmonics. This result extends the concept of conventional metasurfaces, i.e. the equivalence between the modulation on phase in the spatial domain and the change of the wave vector of reflected/transmitted waves [1], to the time domain. In this work, the modulation on phase in the time domain is equivalent to providing an effective additional frequency to the generated waves.

As an important application, they explored the possibility of a wireless communication system via the proposed metasurface. A binary frequency-shift keying (BFSK) communication system was established, in which two harmonics generated from the metasurfaces were employed to represent the information bits 0 and 1 that can be recognized at the receiver end. The giant non-linearity in DCM could also potentially facilitate wave-mixing experiments, as well as photon-pair generation in quantum communication.

Apart from communications, the shifted frequency can be employed to mimic the Doppler frequency of moving objects, leading to potential applications like velocity illusion or distance deception. The results confirm the feasibility of non-linear manipulations with metasurfaces with high efficiency. This design can be potentially applied to higher frequencies or even the visible optical frequency if the unit size can be further reduced and new modulation mechanisms such as phase changing can be used, showing the new possibility of designing non-linear flat optics devices such as optical switches, optical lenses, holography displays, invisible cloaking devices, and LIDAR.
